# White matter changes in Alzheimer’s disease: a focus on myelin and oligodendrocytes

**DOI:** 10.1186/s40478-018-0515-3

**Published:** 2018-03-02

**Authors:** Sara E. Nasrabady, Batool Rizvi, James E. Goldman, Adam M. Brickman

**Affiliations:** 1 Department of Psychiatry, Columbia University, New York, NY USA; 2 Department of Pathology and Cell Biology, Columbia University, New York, NY USA; 3 Department of Neurology, Columbia University, New York, NY USA; 4 The Taub Institute for Research in Alzheimer’s Disease and the Aging Brain, Columbia University, New York, NY USA

**Keywords:** Alzheimer’s disease, White matter, Myelin, Oligodendrocyte, Neurodegeneration, Oxidative stress

## Abstract

Alzheimer’s disease (AD) is conceptualized as a progressive consequence of two hallmark pathological changes in grey matter: extracellular amyloid plaques and neurofibrillary tangles. However, over the past several years, neuroimaging studies have implicated micro- and macrostructural abnormalities in white matter in the risk and progression of AD, suggesting that in addition to the neuronal pathology characteristic of the disease, white matter degeneration and demyelination may be also important pathophysiological features. Here we review the evidence for white matter abnormalities in AD with a focus on myelin and oligodendrocytes, the only source of myelination in the central nervous system, and discuss the relationship between white matter changes and the hallmarks of Alzheimer’s disease. We review several mechanisms such as ischemia, oxidative stress, excitotoxicity, iron overload, Aβ toxicity and tauopathy, which could affect oligodendrocytes. We conclude that white matter abnormalities, and in particular myelin and oligodendrocytes, could be mechanistically important in AD pathology and could be potential treatment targets.

## Introduction

Alzheimer’s disease (AD) is conceptualized as a progressive consequence of two hallmark pathological changes: extracellular neuritic plaques, which are composed of amyloid-beta (Aβ) surrounded by dystrophic neuritic processes, and neurofibrillary tangles, which are intraneuronal aggregates of insoluble cytoskeletal elements, composed mainly of phosphorylated tau protein. These pathological changes are believed to result in neurodegeneration, which can be appreciated with structural neuroimaging as regional and global atrophy [73]. Because of the distribution of this pathology and its associated neurodegeneration, AD is typically considered a disease of the brain’s grey matter. However, over the past several years, neuroimaging studies have implicated micro- and macrostructural abnormalities in white matter in the risk and progression of AD, suggesting that in addition to the neuronal loss characteristic of the disease, white matter degeneration and demyelination may be important pathophysiological features. Myelin loss and the inability of the oligodendrocytes, the cells responsible for the production and maintenance of myelin, to repair myelin damage may be additional central features of AD [5, 53, 55, 60]. Because of the essential role of oligodendrocyte cell lineage in myelin production and remyelination processes, changes in the number of oligodendrocytes or their precursor cells and/or their dysfunction can affect myelin integrity and therefore be potentially implicated in AD pathogenesis.

The purpose of this review is to discuss the evidence for white matter abnormalities in AD with a focus on myelin damage and oligodendrocyte lineage cells and to review the relationship between white matter changes and the pathological hallmarks of AD. In addition, we discuss whether white matter changes are a secondary result of cortical AD pathology or whether they contribute directly or indirectly to the pathogenesis and clinical manifestation of AD.

### Evidence of white matter abnormalities from imaging studies

The observation that neuroimaging-defined white matter abnormalities are characteristic of AD is relatively new. Work from our laboratory, for example, demonstrated that the burden of white matter hyperintensities (WMH), distributed signal abnormalities visualized on T2-weighted magnetic resonance imaging (MRI), predicts incident AD [18, 19, 21], the rate of cognitive decline among individuals with prevalent AD [78], and is associated with genetic risk factors for late onset AD [20]. We recently showed in the Dominantly Inherited Alzheimer’s Network that WMH volume is elevated among individuals with autosomal dominant, fully penetrant mutations for AD up to 20 years before the expected onset of symptoms, demonstrating that white matter abnormalities are indeed a core feature of AD. Furthermore, the appearance of WMH in these patients emerges contemporaneously with AD-related cerebrospinal fluid (CSF) amyloid and tau abnormalities [49]. WMH severity also correlates with CSF levels of Aβ1–42 in preclinical AD [49] and predicts increasing CSF tau levels in individuals with mild cognitive impairment [79]. White matter hyperintensity severity is associated with cerebrospinal fluid (CSF) amyloid levels independent of vascular risk factors [71].

The important role of vascular disease in the development of white matter damage should be emphasized. White matter hyperintensities tend to be distributed in brain areas with relatively low perfusion levels, particularly in the deep, periventricular white matter. The density of vessels in these areas decreases both with normal aging and in AD [23], consistent with reports of decreased blood flow to white matter [69], which could cause hypoxic/ischemic damage in these areas. White matter hyperintensities are related to small vessel disease, and inflammation [26, 61, 64], but comprehensive analysis of postmortem tissue in areas known to be affected by WMH, including evaluation of possible hypoxic damage to oligodendrocyte lineage cells, has not been completed. A recent study reported that parietal WMH pathogenesis in AD is related to axonal loss, through Wallerian-like degeneration, which corresponds to cortical phosphorylated tau burden, and demyelination in patients with AD but to vasculopathy and ischemia (by sclerotic index as a marker of small vessel disease and myelin associated glycoprotein to proteolipid protein ratio as a measure of hypoperfusion) in individuals without AD, suggesting that some degree of WMH is secondary to neurodegeneration in the context of AD [57]. However, neuroimaging studies showed that white matter networks are defective in preclinical AD, at a time when neurodegenerative changes, cortical atrophy, or cortical glucose reduction were not apparent [33]. Vascular and blood brain barrier (BBB) impairments, small hemorrhagic lesions and buildup of iron have been reported in the brains of AD patients even in the preclinical stages of the disease [86].

### Histopathological evidence of white matter abnormalities

**Fig. 1:**
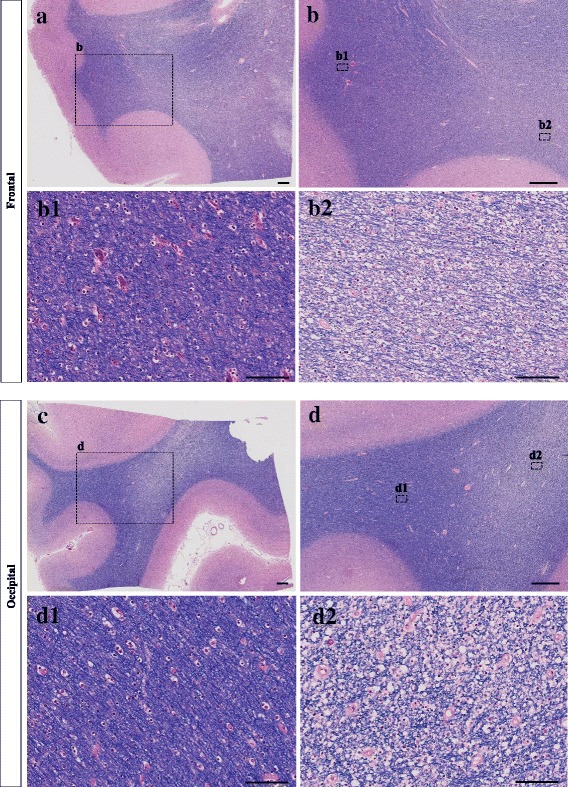
This figure demonstrates examples of white matter myelin loss in brain sections from a patient with Alzheimer’s disease. Tissues from frontal and occipital areas are stained with Luxol fast blue-hematoxylin and eosin (LHE). b2 and b1 represent the regions with and without myelin pallor in the frontal area, respectively. d2 and d1 represent the regions with and without myelin pallor in the occipital area, respectively. The scale bars in images **a**, **b**, **c** and **d** are 1000 μm. The scale bars in images *b1*, *b2*, *d1* and *d2* are 100 μm

The neuroimaging studies establish that there is some degree of white matter abnormality in the context of AD, which may be radiological manifestations of more widespread white matter pathological abnormalities. White matter hyperintensities have been associated histopathologically with myelin pallor, myelin loss [31, 38] (as shown in Fig. 1) and the loss of myelinated axons, as well as changes in arterial adventitia in deep white matter [68]. In our aim to review the widespread white matter abnormalities, we focus on the changes in the myelin sheath and oligodendrocyte lineage cells.

### Myelin damage

In the normal development of the central nervous system (CNS), different brain regions are myelinated at different times. Myelination begins in the fourth month of human embryonic development and continues until the third or fourth decade of life [22, 47] . In general, the spinal cord and brain stem myelinate earlier, while other areas, such as the telencephalon, the entorhinal cortex, hippocampus and the amygdala myelinate later [17, 30]. Diffusion tensor imaging studies show the development of association tracts in post-adolescent subjects [47]. In addition, there is a particular susceptibility for demyelination in areas that are myelinated at older ages, a phenomenon referred to as “neuropathologic retrogenesis” [8, 12, 17, 66, 76]. Myelin loss has been observed consistently in AD and the later-myelinated areas are also most vulnerable [13, 36, 65]. They demonstrate significantly greater myelin loss compared with areas that myelinate earlier [6]. Analysis of postmortem brain tissue of AD patients has revealed that the white matter is altered chemically, compared with that of patients without dementia: the amounts of total protein, myelin basic protein (MBP), myelin proteolipid protein (PLP), Cyclic nucleotide phosphohydrolase (CNPase), and cholesterol is significantly decreased, indicating a loss of myelin. White matter fatty acid ratios are also altered in AD [67].

White matter and myelin changes in AD need to be taken with respect to changes during aging. For example, the overall hemispheric white matter volume decreases with age [50]. How much of this is due to changes in water content or water infusibility is not completely clear [42]. However, a decrease in the total length of myelinated fibers, reaching a 45% decrease from 20 to 80 year-old individuals and the appearance of thinner axons has been reported [50]. Thus, a number of investigations conclude that with age, myelin production by oligodendrocytes continues but leads to thinner myelin sheaths and shorter internodes [50]. Axon caliber decreases in experimental models of demyelination and remyelination [52]. Thinner myelin sheaths and smaller axons can lead to functional white matter deficits due to conduction failure and by a greater vulnerability to trauma, oxidative stress, or Aβ toxicity [5]. Axonal loss and demyelination are both associated with white matter abnormalities in AD and are predictors of severity of white matter abnormalities [57]. Future studies with the aim of preventing or repairing myelin damage could elucidate the impact of white matter changes as one of the core pathologies of AD.

### Abnormalities of oligodendrocyte lineage cells in AD

**Fig. 2:**
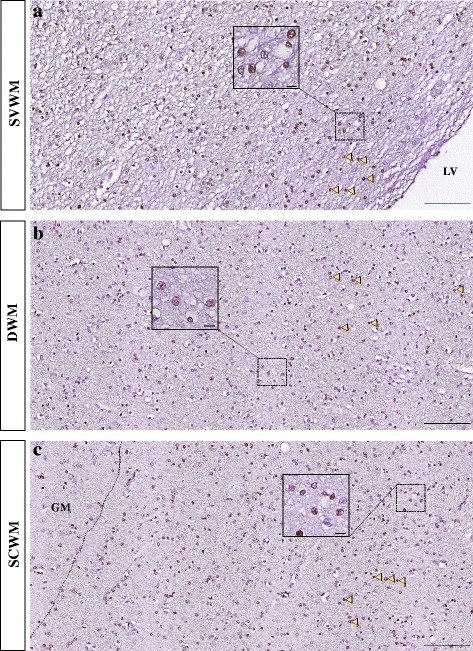
An example of Olig2+ oligodendrocyte distribution throughout the white matter from a neurologically-healthy adult, postmortem brain. The insets show Olig2+ nuclei at higher magnification. H&E counterstaining. Arrowheads: Olig2+ nuclei (brown). Dashed line: the border of white and grey matter. LV: lateral ventricle; SVWM: subventricular white matter; DWM: deep white matter; SCWM: subcortical white matter; GM: grey matter. The scale bars in **a**, **b** and **c** are 100 μm and the scale bars in the insets are 10 μm

The primary role of oligodendrocytes is to produce myelin, but they also play a supportive, modulatory and regulatory role for neurons, including the production of neurotrophic factors, inhibition of neurite growth, and stabilization of neuronal connectivity [44, 70]. The adult central nervous system contains both precursor cells for oligodendrocytes in addition to mature, myelinating cells [10]. Oligodendrocyte precursor cells (OPCs) in the adult central nervous system are capable of proliferating and migrating and effecting new myelination after demyelinating insults. Animal studies have shown that during adulthood, oligodendrocytes are generated from ventricular-subventricular zone and the new oligodendrocytes progenitors migrate to white matter tracks from there [59]. Mature human oligodendrocytes in the condition of being deprived from their myelin sheath may return into their previous subtype capacity and change their phenotype and their myelination program [37]. The different oligodendrocyte lineage cells express changes in morphology during their development and maturation [10]. Figure 2 shows an example of oligodendrocyte distribution throughout white matter areas in an adult postmortem human brain. In mammals, OPCs, by presence of intrinsic hypoxia-inducible factor (HIF) signaling, control white matter angiogenesis, axonal integrity, and the onset of myelination at postnatal stages [85]. Oligodendrocytes are able to modulate ion homeostasis in the axon environment [60]. OPCs are the specific glial cells that directly make synapses with neurons; they build synapses with glutamatergic neurons in the hippocampus (rat) [15], cortex (mouse) [24], white matter tracks (rat) [46] and other areas. However, they only make postsynaptic connection with neurons. The behavior of OPCs can be controlled by neurotransmitters and by surrounding neuronal demands. For example, in the presence of higher neural activity, oligodendrocytes change the amounts of myelin sheaths, which affects the electrical transmission in the neuronal network [32]. We could speculate that neuronal AD pathology, which is characterized by neuronal and axonal dysfunction, could alter the amounts of myelin produced by oligodendrocytes. This dynamicity of myelin and oligodendrocytes thus could ultimately affect behavior [58].

A number of human and animal studies investigated oligodendrocyte changes in AD. In one study, 6–8-month-old APPPS1 mice showed an increase in the numbers of OPCs, while the number of Olig2+ cells in postmortem tissues of AD patients was decreased [11]. Another study reported a higher number of MAP-2 positive remyelinating oligodendrocytes in the areas adjacent to periventricular white matter lesions and a higher number of PDGFR-α positive OPCs in white matter lesions [74]. In a PS1 knock-in mouse model, increased vulnerability and death of oligodendrocytes due to glutamate and Aβ were demonstrated and these cells showed a deficit in calcium regulation [60]. This study suggests that the abnormalities of oligodendrocytes in the presence of a PS1 mutation could be an early event in the disease course. Another study demonstrated that MBP and the number of myelinating oligodendrocytes were decreased in 6-month old triple transgenic mice (3xTg-AD), the number of immature oligodendrocytes remained unchanged and mature non-myelinating cells were increased [29]. The authors reported that myelinating oligodendrocytes are highly sensitive to oxidative stress due to their higher metabolic demand, higher iron, and lipid content. Reduction of the diameter of oligodendrocyte nuclei in AD patients in parahippocampal white matter was also reported [35] while the mean nuclear diameter of neurons remained unchanged. Alzheimer’s-related changes in oligodendrocytes for human studies and animal models are summarized in Table 1.

**Table 1:** This table summarizes the studies, the specimen that was used, and oligodendrocyte alterations in AD animal models and human

The model and specimen	Oligodendrocyte changes			Source
PS1 knock-in mouse	Vulnerability and death of OLs			Pak et al. 2003
Postmortem AD	Increased MAP-2 positive remyelinating OLs adjacent to WM lesions	Increased PDGFR- α positive OPCs in WM lesions	No change in Myelinating OLs in deep white matter	Simpson et al. 2007
3xTg-AD mouse	Decreased myelinating OLs	No change in immature OLs	Increased mature non-myelinating OLs	Desai et al. 2010
Postmortem AD	Reduced OLs nuclear diameter in parahippocampal white matter			Gagyi et al. 2011
APPPS1 mouse	Increased OPCs number			Behrendt et al. 2013
Postmortem AD	Decreased Olig2+			Behrendt et al. 2013

It depicts the verity of the results in different animal models and human studies. *PS1* Presenelin-1, *OL* oligodendrocyte, *MAP* microtubule associated protein, *PDGFR* platelet-derived growth factor receptor, *OPCs* oligodendrocyte progenitor cells, *WM* white matter, *3xTg-AD* triple transgenic AD mouse model. *APPPS1 mouse* mouse with both APP and PS1 transgenes

One of the frequently accepted themes related to oligodendrocyte damage in AD is that these cells suffer from *oxidative stress*, which can be produced by a wide range of factors. The adult CNS contains oligodendrocyte precursor cells, which can be mobilized to differentiate into myelinating oligodendrocytes [10]. Oxidative stress impairs the differentiation of OPCs, in part by decreasing the levels of expression of genes that promote oligodendrocytes differentiation, such as Shh, Sox10 and HDAC3 [34]. In cell culture, pre-oligodendrocytes show a sensitivity to oxidative stress and glutathione depletion [2]. In rat cell cultures, low antioxidant content and high iron capacity, in addition to excitotoxicity through metabotropic glutamate receptors, makes oligodendrocytes more vulnerable to oxidative stress [27, 77]. Other factors affecting oligodendrocytes are listed below.

*Aβ and tau*: Several studies suggest that Aβ is toxic to oligodendrocytes [28, 29, 41, 48, 84]. For example, a cell culture study of rat oligodendrocytes demonstrated that Aβ-induced oxidative stress can drive oligodendrocyte death and dysfunction [84]. This study also showed that mitochondrial DNA damage and the consequent NF-kB and AP-1 activation are other possible mechanisms of Aβ toxicity for oligodendrocytes [84]. Note that although amyloid plaques are exceedingly rare in AD white matter, the levels of soluble Aβ are elevated in the white matter [25]. Thus, a direct exposure of white matter oligodendrocytes to increased amounts of Aβ is likely. Although there are toxic effects of Aβ on oligodendrocytes, clinical trials that have aimed to remove the Aβ plaque in symptomatic AD patients, did not prevent the progressive neurodegeneration and cognitive decline in AD patients [39, 72]. These findings suggest that this toxic effect needs to be targeted earlier or it could not be the only pathology leading to cell death and atrophy in symptomatic patients. In addition to the effects of amyloid pathology, the impact of tau pathology on white matter needs to be considered. Tau can affect the normal function of neurons through a toxic gain of function or a loss of its normal function in stabilizing microtubules. Although severe neocortical tauopathy occurs in later stages of AD and mostly affects grey matter, phosphorylated tau transforms into neurofibrillary tangles in neurons as well as glial tangles in astrocytes or oligodendroglia [4]. Furthermore, phosphorylated tau in grey matter is associated with white matter abnormalities and demyelination in AD patients [56, 57]. The increased levels of calpain2 in the AD white matter, an indicator of axonal loss, was shown to be associated with increased cortical phosphorylated tau and amyloid [57] and the phosphorylated tau showed to be a predictor for white matter hyperintensities [56].

*Iron*: During myelination, oligodendrocytes require 2–3 fold higher energy levels than other cell types in the CNS to produce such an extensive amount of membrane. Oligodendrocytes synthesize cholesterol, a process that is highly metabolically demanding, making them vulnerable to hypoperfusion, excitotoxicity, heavy metals, and free radicals that induce oxidative stress. Oligodendrocytes have the highest iron content of all cell types, which increases with age and even further in AD [5]. Oligodendrocytes at all stages of their differentiation, compared with other glial cells contain smaller amounts of antioxidant agents (e.g. glutathione peroxidase) and only half of the glutathione reductase activity [43]. Thus, a high iron content and a low antioxidant content make oligodendrocytes one of the most vulnerable cell classes to oxidative stress in the CNS. If oxidative stress is exacerbated by age, it may lead to increased cell damage [81]. Bartzokis by comparing a map of cortical myelination with maps of Aβ deposition hypothesized that age-associated myelin breakdown leads to iron release from oligodendrocytes and that this iron release promotes Aβ oligomerization in the parenchyma [7].

*Hypoxia/Ischemia*: Deep white matter areas lie at the ends of the CNS arterial circulation, making them susceptible to decreases in blood flow and oxygenation. Some anterior and posterior white matter lies in watershed zones between the anterior cerebral and middle cerebral arteries and middle cerebral and posterior cerebral arteries respectively. Vascular pathology in these regions is greater in patients with AD than in individuals without dementia [23, 69]. Late-stage oligodendrocyte progenitors are more sensitive to hypoxic or ischemic damage than early-stage progenitors and more mature oligodendrocytes [3]. A recent rodent study, using single cell RNA sequencing, identified a population of oligodendrocyte precursors as vascular and leptomeningeal cells. These OPCs are located along vessels and they show similarities with pericyte lineage cells [51]. In addition, to emphasize the importance of relationship between vascular system and oligodendrocyte lineage cells Tsai showed that those OPCs require the physical infrastructure provided by the vascular system to facilitate their migration during development [80].

*Excitotoxicity*: In general, oligodendrocytes show a great vulnerability to excessive ATP and/or activation of glutamate receptors [55]. Oligodendrocytes express a wide variety of receptors and membrane channels (e.g. ionotropic glutamate and ATP receptors, ligand gated Ca^2+^ channels and P2x7 receptors). Because of the lack of the GluR2 subunit in oligodendrocyte AMPA receptors, there is a higher permeability to Ca^2+^ ions compared with neurons [54]. Another example is the sustained activation of p2x7 receptors in oligodendrocytes due to excitotoxicity or to high levels of ATP/ADP/AMP, which leads to excessive Ca^2+^ in the cytosol and the activation of apoptosis through caspase-3 activation. Extensive activation of these receptors can result in oligodendrocyte damage and subsequently myelin destruction. Back and colleagues showed maturation-dependent vulnerability of oligodendrocytes caused by intracellular glutathione depletion [2]. In addition, as we mentioned, oligodendrocytes and myelin damage due to excitotoxicity and calcium dysregulation could be an early pathological feature of AD [45, 60].

*DNA damage*: Age related DNA damage in myelinating oligodendrocytes may contribute to myelin loss [81, 82]. Postmortem analysis of white matter lesions obtained from aging individuals shows the presence of oxidative damage (8-OHdG immunoreactivity) in oligodendrocyte nuclear DNA. These cells are also positive for senescence markers such as SA-β-gal [1]. In older adults, excessive DNA damage occurs in vulnerable oligodendrocytes and the DNA repair mechanism becomes overwhelmed. Studies of changes in genomic integrity and genomic instability of oligodendrocytes in the white matter of patients and animal models could illuminate the role of oligodendrocyte in white matter damage and pathology of AD [81, 82]. In addition, oligodendrocyte lineage transcription factor 2 (Olig2) is located on chromosome 21 which is 6.8 Mb telomeric of the amyloid precursor protein (APP) gene. The possibility of these two genes interacting in a context of the disease needs to be studied [75].

## Discussion

**Fig. 3:**
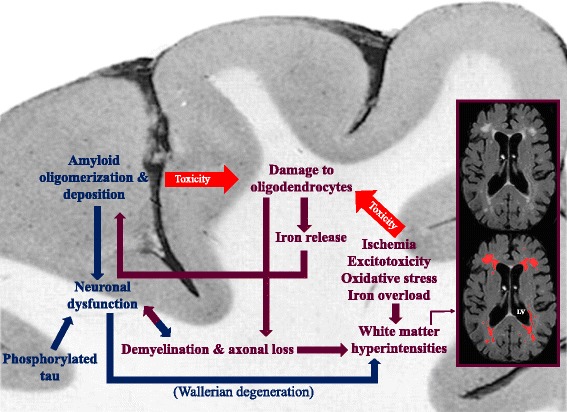
This figure summarizes the pathological cascades, and their relation with each other, occurring during the development of Alzheimer’s disease in white matter and cortex. While ischemia, excitotoxicity, oxidative stress, and iron overload in white matter damage oligodendrocytes, on one hand, and amyloid toxicity affects them, on the other hand, the iron released from damaged oligodendrocytes promotes amyloid polymerization and deposition in grey matter. The consequent demyelination and axonal loss result in further white matter damage and neuronal dysfunction. Neuronal dysfunction is also a result of amyloid deposition in cortex and a proposed cause for white matter abnormalities in AD patients. White matter hyperintensities are labelled with red in the MRI (FLAIR) scan of an AD patient. Blue arrows: direction of the damages originating in grey matter. Maroon arrows: direction of the damages originating in white matter. LV lateral ventricle

A variety of structural, histopathological and biochemical pathologies take place in the white matter of AD patients (summarized in Fig. 3). In this review, we have tried to answer two questions:

*What changes occur in white matter in the course of AD and what is the relationship between these changes and the pathological hallmarks of the disease?*

Radiological, pathological, and molecular changes occur in the white matter of AD patients. Radiological markers of white matter damage occur as early as 22 years before the estimated age of symptom onset in humans who carry AD mutations [49]. These white matter changes are believed to reflect demyelination and axon damage [63]. It is possible that the oligodendrocytes or the precursors responsible for remyelination of these areas are altered in number and in DNA stability or are functionally less efficient in the presence of genetic changes, oxidative stress, increased iron levels, and vascular pathology [5, 81]. In addition to gross white matter damage in AD, there are chemical alterations marked by loss of proteins and cholesterol. The decreases in the levels of myelin proteins, such as myelin basic protein (MBP), myelin proteolipid protein (PLP) and CNPase, in white matter reflects the changes in oligodendrocytes and myelin sheaths. In animal models of AD, the white matter disruption and changes in myelin marker expression are among the earliest pathological changes [16, 30]. Although white matter changes are believed to be partly related to neuronal degeneration in the cortex [57], there is also evidence that oligodendrocyte and myelin pathology, which are detected in AD mouse models, are affected prior to appearance of amyloid and cortical pathology. It is not clear if these changes are independent of cortical pathology or the cortical structural damages are beyond the detection limits of methods at early stages of the disease [29, 30, 40] and subtle neurodegenerative changes could precede or promote white matter changes even if they are not detectable with conventional methods. In terms of therapeutic implications, contemporary clinical trials have focused on the removal of fibrillar forms of amyloid protein as a primary target, motivated by the “amyloid hypothesis” that has dominated the field’s conceptualization of the disease for many years [73]. Accordingly, interventions that target the removal of amyloid would arrest the progression of disease and improve cognitive outcomes. However, to date, clinical trials aimed to clear Aβ plaques in AD have not resulted in clinical improvement or reduction in the rates of disease progression. New strategies for disease treatment and prevention are therefore necessary. The consistent observations that implicate white matter abnormalities in AD pathogenesis and progression point to opportunities to target potential novel mechanisms implicated in the disease.

*Are the clinical changes in AD secondary only to the cortical hallmarks of the disease or do white matter abnormalities contribute directly or indirectly to the disease?*

Beside amyloid hypothesis as the cause of AD, it is crucial to note that there is a weak association between amyloid plaques and AD symptoms [5]. Further, senile plaques can be found in about 20–40% of older adults without symptoms of dementia and cognitive impairment [14, 62]. As discussed above, myelin loss contributes to cognitive decline in humans [67, 83] and in AD animal models, early changes in white matter are followed by the first cognitive impairment detected after intracellular accumulation of Aβ but before Aβ plaques appear [29]. Altering axonal conduction by demyelination or axonal damage could directly and/or indirectly affect cognition. Future studies with the aim of repairing myelin loss could clarify the impact of white matter changes on AD pathogenesis and may have therapeutic benefit. In addition, dysfunctional oligodendrocytes early in the course of the disease may not be able to play a protective role for neurons and their axons. Hence, the process of remyelination and myelin repair will be affected. Signal conductivity, and synchronicity of impulses, which are required for information processing between neurons, depend on the amount of myelin produced by oligodendrocytes and therefore will be affected [10]. In general, a number of animal AD model studies suggest that white matter pathology emerges prior to appearance of cortical plaques and tangles [29, 30, 40]. Human studies with autosomal dominant forms of AD suggest that Aβ levels begin to change as early as 25–30 years prior to symptom onset, followed by tau pathology about 15 years prior to symptom onset, cerebral hypometabolism, brain atrophy, and cognitive and functional deficits [9, 29]. As noted above white matter abnormalities are also early findings in these patients [49], but causal relationships among AD biomarkers in humans are difficult to infer even from observed temporally-ordered observations; white matter changes appear to emerge contemporaneously with other AD pathology, but it is unclear if one is causing the other. Studies of 3xTg-AD triple transgenic AD mice show that the first pathological features start to appear in 2 to 6-month old mice as white matter disruption and changes in myelin marker expression in the hippocampus and entorhinal cortex. At this age, the mice do not show learning or memory deficits [16, 30]. Cognitive impairment emerges after at around 3 to 6-months of age [29]. The studies discussed regarding vascular insufficiency in white matter suggest that the white matter pathology is not caused by cortical pathology. On the other hand, neuronal accumulation of tangles and neuronal death will lead to axonal loss in white matter [57]. It is not clear how this would produce white matter hyperintensities in deep white matter, however, it could well contribute to a more widespread decrease in axons and myelin.

In conclusion, the various observations suggest that white matter abnormalities and in particular impaired myelin and oligodendrocytes could promote cognitive impairment and AD pathology and could be the important targets for studying and early treatments of AD. Future studies with the aim of repairing myelin damage, beside other efforts focused on the hallmark of Alzheimer’s disease, could elucidate the impact of white matter changes as one of the core pathologies of AD.

## Abbreviations

3xTg-AD: triple transgenic AD mice model
AD: Alzheimer’s disease
AP-1: activator protein 1
ATP: adenosine triphosphate
Aβ: amyloid-beta
BBB: blood brain barrier
CNPase: cyclic nucleotide phosphohydrolase
CNS: central nervous system
CSF: cerebrospinal fluid
HDAC3: histone deacetylase 3
HIF: hypoxia-inducible factor
MBP: myelin basic protein
MRI: magnetic resonance imaging
NF-kB: nuclear factor kappa-light-chain-enhancer of activated B cells
OPC: oligodendrocyte precursor cell
P2x7 receptors: P2X purinoceptor 7
PLP: myelin proteolipid protein
Shh: sonic hedgehog
Sox-10: SRY-related HMG-box
WMH: white matter hyperintensity
